# Lineage Tracing of Resident Tendon Progenitor Cells during Growth and Natural Healing

**DOI:** 10.1371/journal.pone.0096113

**Published:** 2014-04-23

**Authors:** Nathaniel A. Dyment, Yusuke Hagiwara, Brya G. Matthews, Yingcui Li, Ivo Kalajzic, David W. Rowe

**Affiliations:** 1 Department of Reconstructive Sciences, College of Dental Medicine, University of Connecticut Health Center, Farmington, Connecticut, United States of America; 2 Department of Biology, College of Arts and Sciences, University of Hartford, Hartford, Connecticut, United States of America; University of Minnesota Medical School, United States of America

## Abstract

Unlike during embryogenesis, the identity of tissue resident progenitor cells that contribute to postnatal tendon growth and natural healing is poorly characterized. Therefore, we utilized 1) an inducible Cre driven by alpha smooth muscle actin (SMACreERT2), that identifies mesenchymal progenitors, 2) a constitutively active Cre driven by growth and differentiation factor 5 (GDF5Cre), a critical regulator of joint condensation, in combination with 3) an Ai9 Cre reporter to permanently label SMA9 and GDF5-9 populations and their progeny. In growing mice, SMA9+ cells were found in peritendinous structures and scleraxis-positive (ScxGFP+) cells within the tendon midsubstance and myotendinous junction. The progenitors within the tendon midsubstance were transiently labeled as they displayed a 4-fold expansion from day 2 to day 21 but reduced to baseline levels by day 70. SMA9+ cells were not found within tendon entheses or ligaments in the knee, suggesting a different origin. In contrast to the SMA9 population, GDF5-9+ cells extended from the bone through the enthesis and into a portion of the tendon midsubstance. GDF5-9+ cells were also found throughout the length of the ligaments, indicating a significant variation in the progenitors that contribute to tendons and ligaments. Following tendon injury, SMA9+ paratenon cells were the main contributors to the healing response. SMA9+ cells extended over the defect space at 1 week and differentiated into ScxGFP+ cells at 2 weeks, which coincided with increased collagen signal in the paratenon bridge. Thus, SMA9-labeled cells represent a unique progenitor source that contributes to the tendon midsubstance, paratenon, and myotendinous junction during growth and natural healing, while GDF5 progenitors contribute to tendon enthesis and ligament development. Understanding the mechanisms that regulate the expansion and differentiation of these progenitors may prove crucial to improving future repair strategies.

## Introduction

Understanding the origin of resident tendon progenitors and the factors that influence their differentiation are critical to designing novel repair strategies. During development, tendon progenitors originate in the sclerotome and express the basic-helix-loop-helix transcription factor scleraxis (Scx) [1]. Scx+ cells contribute to the tendon midsubstance as it condenses between the adjacent muscle and cartilage. Sox9, a SRY-related transcription factor which is important in cartilage differentiation, is co-expressed with Scx in cells that eventually give rise to the tendon fibrocartilage within the enthesis [2], [3]. Sox9+ cells beneath this layer contribute to the underlying bone while the adjacent muscle is derived from the myotome. While our understanding of the origin of progenitors that give rise to tendon in the embryo is improving, little is known about the anatomical origin of resident progenitors within the tendon that contribute to these regions during postnatal growth, how these cells proliferate and expand in 3D space during growth, and whether these cells also contribute to adult natural healing following injury.

Recent studies have begun to identify and characterize the progenitor niche within tendon. Bi et al showed that the small leucine rich proteoglycans fibromodulin and biglycan contribute to this niche [4]. Others have suggested that perivascular progenitors exist within the tendon midsubstance and paratenon [5], [6]. In addition, researchers are beginning to isolate cells from tendon that express stem/progenitor markers, show multi-potency in vitro, and improve tendon repair in vivo [7], [8]. However, detailed in vivo lineage tracing of tendon progenitors demonstrating their expansion potential during growth and their reparative potential following injury has not been pursued in great detail.

Alpha smooth muscle actin (αSMA), while highly expressed in smooth muscle cells within blood vessel walls, is also a marker for a mesenchymal progenitor that contributes to bone, fat, and perivascular lineages [9]–[12]. αSMA is highly expressed in early stages of primary bone marrow stromal cultures and αSMA+ progenitors within the stromal compartment contribute to trabecular and endocortical bone formation while progenitors within the periosteum contribute to callus formation during fracture healing [9]. αSMA progenitors within the periodontium contribute to the periodontal ligament and cellular cementum during growth and may have a perivascular origin [13]. While αSMA is expressed by myofibroblasts during early tendon healing [14], it is unclear whether αSMA can identify progenitors within tendon.

Growth differentiation factor 5 (Gdf5) is a crucial regulator of joint development and Gdf5+ progenitors contribute to the formation of intra-articular structures including articular cartilage, ligaments, fibrocartilage, and synovial lining [15]. Gdf5 deficiency also delays tendon healing [16] but little is known whether Gdf5 regulates tendon development and maturation. The tendon enthesis forms in a modular fashion from Scx and Sox9 progenitors within bone eminences near joints that do not arise from the primary cartilage [3]. In fact, these Scx/Sox9 co-expressing cells likely originate from the GDF5+ interzone and extend to the lateral edges of the joint to form the eminences and tendon attachments.

The objective of this study is to identify and characterize the expansion of tissue resident tendon progenitors that contribute to normal cell turnover during growth and natural healing during adulthood. Through detailed lineage tracing of SMA9+ progenitor cells, we found that SMA9+ cells in the tendon midsubstance are an amplifying progenitor population during growth. These cells do not contribute to the fibrocartilage within the tendon enthesis and ligamentous cells within the knee, which originate from a Gdf5 lineage. Finally, SMA9+ progenitors in the paratenon are the main contributors to the healing response following injury in the adult patellar tendon.

## Materials and Methods

### Ethics Statement

This study was carried out in strict accordance with the recommendations in the Guide for the Care and Use of Laboratory Animals of the National Institutes of Health. The protocol was approved by the Institutional Animal Care and Use Committee of the University of Connecticut Health Center (Protocol Number: 100547-1015). All surgery was performed under isoflurane anesthesia, and all efforts were made to minimize suffering.

### Transgenic Mice

#### Two Cre driver transgenic mice were utilized in this study

**αSMACreERT2.** The tamoxifen-inducible αSMACreERT2 mice, which label a mesenchymal progenitor cell population, were described previously [9]. GDF5Cre: The constitutively active GDF5Cre mice were shown previously to contribute to several intra-articular structures [17].

#### Lineage tracing combinations

The Cre driver mice were crossed with the Ai9 Cre reporter mouse line [18] (Jackson Labs), which contains a loxP-flanked STOP cassette that prevents transcription of CAG promoter-driven expression of tdTomato fluorescent protein, resulting in αSMACreERT2-Ai9 (SMA9) and GDF5Cre-Ai9 (GDF5-9) mice. In this strategy cell populations that are the progeny of the cells that expressed the Cre driver at the time of tamoxifen administration (SMA9) or at anytime in the animal’s life (GDF5-9) will express the red fluorescent signal of Ai9 due to Cre-mediated excision of the STOP cassette.

#### Tendon and ligament marker

Along with anatomical location, ScxGFP mice were used to clearly define cells within tendon and ligaments [19], [20]. These animals were crossed with the SMA9 mice to generate a triple transgenic animal (SMA9-ScxGFP) that can trace a progenitor cell into a cell type within the tendon and ligament lineages.

A total of 52 transgenic mice were used for this study, including 22 and 30 transgenic mice for the growth and injury studies, respectively.

### Lineage Tracing during Tendon Growth

Two intraperitoneal injections of tamoxifen (Sigma Aldrich) were delivered on consecutive days to 3–4 week old SMA9 or SMA9-ScxGFP mice at a dose of 75 µg/g body weight. The SMA9 labeled cells were then analyzed on 2, 21, 42, and 70 days following tamoxifen treatment to assess their amplification during growth, characteristic of a resident progenitor population (n = 3–5 per time point). The GDF5-9 mice were analyzed at the ages of P0 (day of birth) and P56 (8 week old) (n = 3 per time point).

### Patellar Tendon Injury

Full-length, full-thickness, central third patellar tendon defects were created in adult SMA9 and SMA9-ScxGFP mice (average age = 23±3 weeks). Following aseptic preparation, two #11 scalpel blades were clamped together with needle holders and used to make longitudinal incisions to create the defect (0.4 mm wide). The central region of the tendon was then excised and the skin was closed with 5–0 nylon sutures. The contralateral limbs were treated as uninjured controls. The animals were allowed free movement within their cages until two photon and histological analysis at 1, 2, and 5 weeks post-injury (n = 5 per time point).

### Histology and Immunohistochemistry

Animals were euthanized via CO_2_ asphyxiation. Limbs were fixed in 10% formalin for 1–2 days at 4°C and then imaged on the two photon microscope. The samples were then transferred to 30% sucrose overnight and embedded in cryomatrix (Thermofisher Scientific). Thin sections (8 µm) were made in several tendons in both the fore and hind limbs using a cryofilm technique (Section-lab, Hiroshima, Japan) [20], [21] on a Leica CM3050S cryostat. Natural healing sections were blocked (Power Block; Biogenex) and stained with anti-tenascin-C (1∶500; Abcam) overnight at 4°C. The slides were incubated with a secondary antibody (Alex Fluor IgG 647; Life Technologies), counterstained with DAPI, and imaged on a Zeiss Imager-Z1 microscope.

### Two Photon Imaging

Formalin fixed limbs were submersed in 1X PBS during imaging on the Prairie Ultima IV multiphoton microscope using a 20X/0.95W Olympus water immersion objective. All fluorophores (GFP and tdTomato) and second harmonic generation (SHG) for collagen were visualized at an excitation wavelength of 890 nm and bandpass filters of 435–485 nm (SHG), 500–550 nm (GFP), and 570–620 nm (tdTomato). Multiple tiled stacks (459×459 µm/tile) were acquired at either 256×256 or 512×512 resolution with z-depth increments of 1.8 µm. Tiled stacks were stitched using the grid/collection stitching plugin [22] and 3D reconstructions were created using the 3D viewer plugin [23] within Fiji image analysis software [24].

### Image Analysis

All image quantification was done using Fiji image analysis software [24].

#### Growth study

The number of SMA9+ cells within the tendon midsubstance were quantified from sagittal sections of the patellar tendon at 2, 21, and 70 days following the last tamoxifen injection. A region of interest was manually drawn to capture the tendon midsubstance without the proximal or distal attachments. Eight-bit grayscale images from the dapi channel were converted to binary images by setting an equivalent threshold across the treatment groups. A watershed was applied to segment nuclei which were touching. The number of nuclei was then counted using the analyze particles function within Fiji. Since the tdTomato fluorescent signal overlays with the cell nucleus, the binary dapi channel was used as a mask to quantify the mean grayscale intensity of the tdTomato signal within the area of each nucleus. The SMA9+ positive cells above a minimum intensity threshold were then quantified and reported as percentage of the total number of cells within the tendon midsubstance.

#### Injury study

The SMA9+ and ScxGFP+ cells were quantified from two photon image stacks at 1, 2, and 5 weeks post-injury. A minimum threshold was applied to each SMA9 and ScxGFP stack to create binary images of positive intensity. The positive area for each individual channel was quantified and the two channels were overlaid to quantify the double positive (SMA9+ ScxGFP) area. The SMA9, ScxGFP, and double positive cell areas were quantified in 3 regions of interest (800 µm long×400 µm wide) at distal, mid, and proximal regions of the defect.

### Statistics

The number of SMA9+ cells in the patellar tendon during growth was analyzed via one-way ANOVA with time post-injection as the fixed factor (p<0.05). The number of SMA9 cells within multiple tendons was also compared at day 21 via one-way ANOVA. Following injury, the area of SMA9, ScxGFP, and double positive cells was analyzed via one-way MANOVA with time post-surgery as the fixed factor (p<0.05).

## Results

### SMA9+ Cells Exist in 4 Distinct Populations in and around Tendons

**Figure 1:**
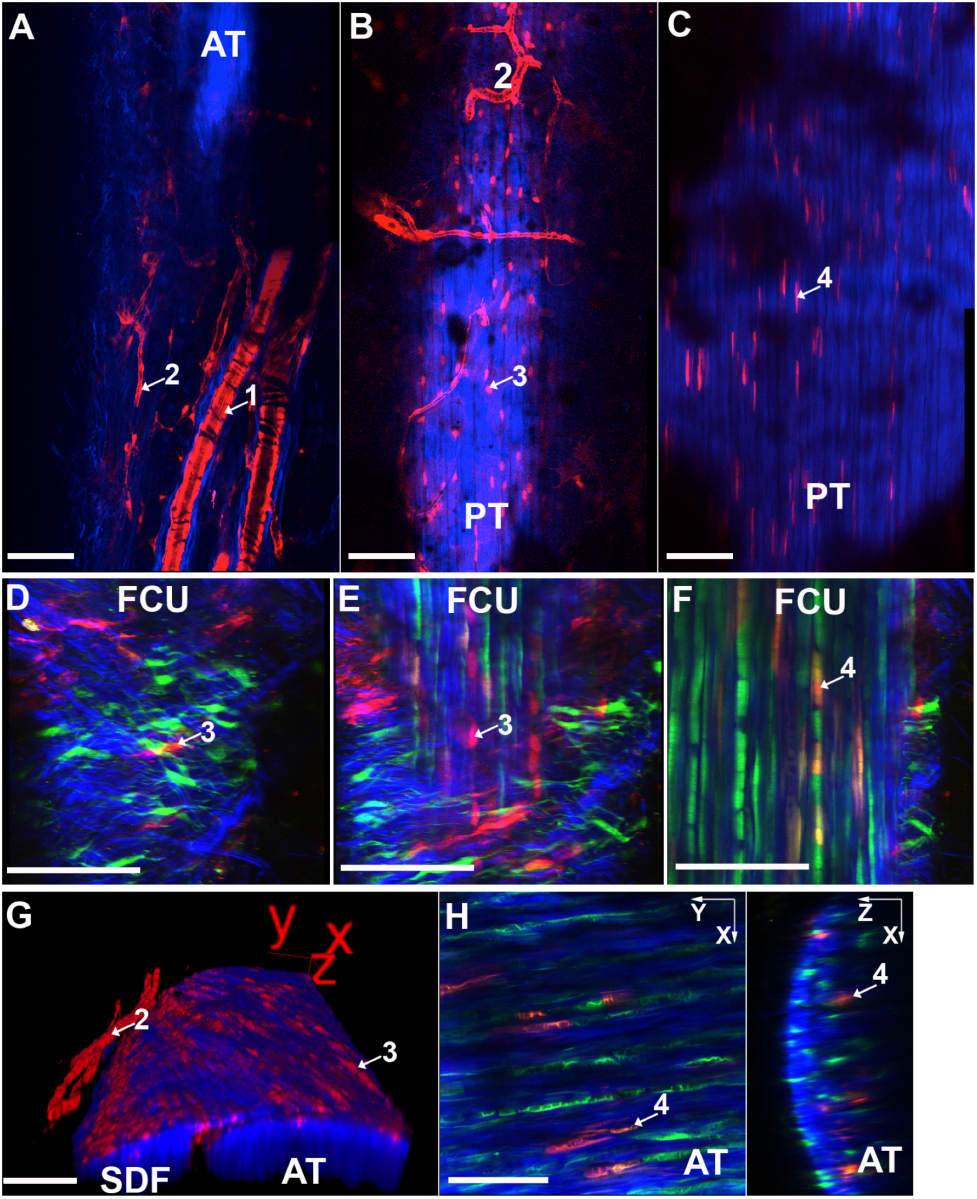
SMA9 model labels 4 distinct cell populations in and around the tendon midsubstance. Following tamoxifen injection, SMA9+ cells (Red) are found in 1) smooth muscle cells in large blood vessels outside of the tendon (A), 2) perivascular cells in smaller vessels (A,B,G), 3) paratenon/sheath/retinacular cells on the tendon surface (B,D,E,G), and 4) tendon fibroblasts within the midsubstance (C,F,H) of the patellar (PT), flexor carpi ulnaris (FCU) tendon, Achilles (AT), and superficial digital flexor tendons (SDF) on 21 days (A–C,G) and 42 days (D–F,H) following injection. Images and 3D reconstructions (G) were created using two photon microscopy in SMA9 (A–C,G) and SMA9-ScxGFP mice (D–F,H). SMA9+ cells within the tendon body are ScxGFP+ (green) (F,H), unlike SMA9+ in the paratenon and tendon sheath (D–E). B&C are optical slices at the tendon surface and 50 µm deep in the PT, respectively. D–F are optical slices at the surface of the sheath, middle of the sheath, and 130 µm deep, respectively. G displays SMA9+ cells in the retinaculum covering the SDF and AT. Blue – second harmonic generation (SHG) for collagen. Scale bars = 100 µm. The authors encourage the readers to view figure S1 for the anatomical location of each panel in this figure.

**Figure 2:**
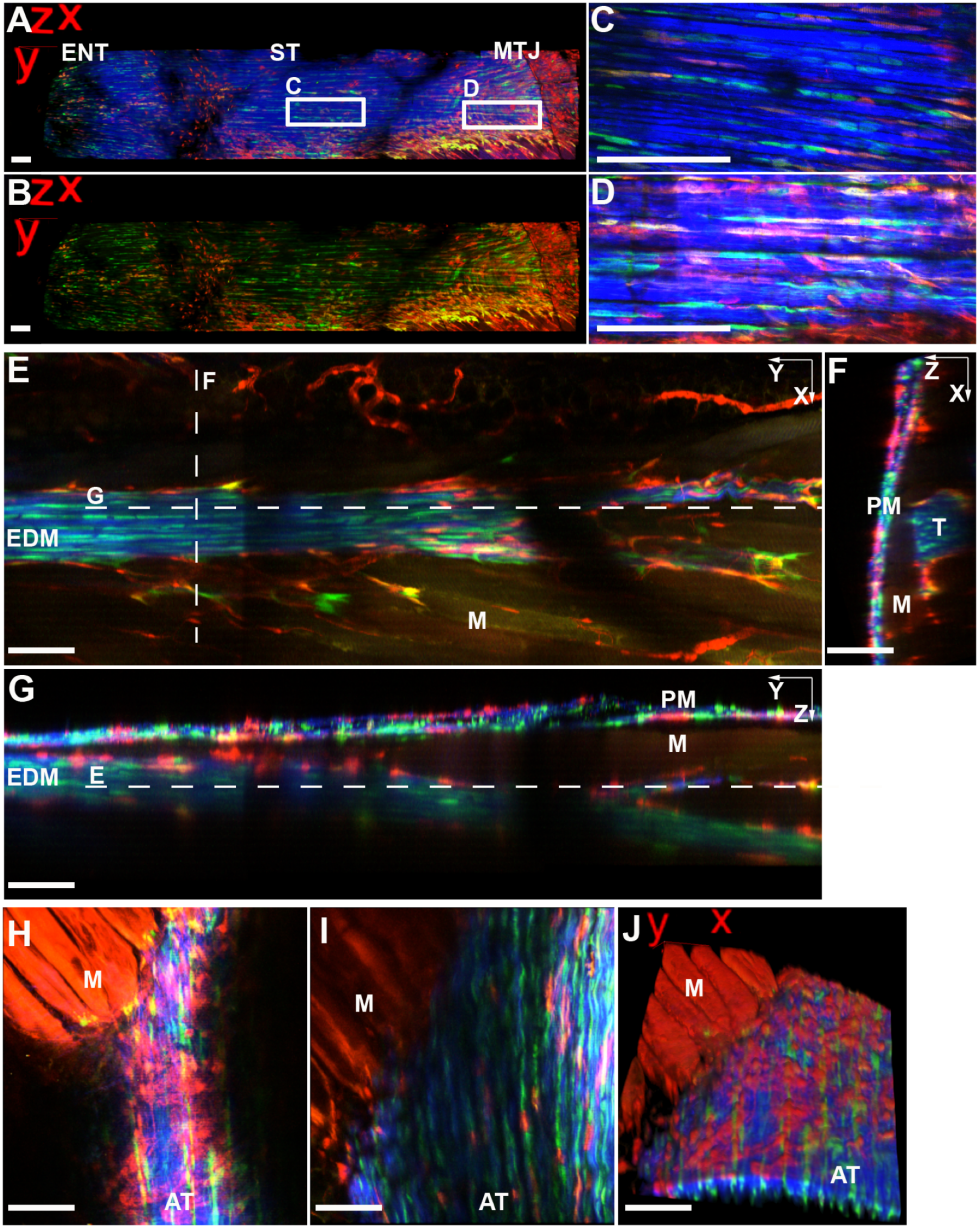
SMA9+ cells are found in the tendon body, perimysium, and muscle fibers at the myotendinous junction. A–D) SMA9+ (red) cells in the supraspinatus tendon (ST) span from the anterior surface near the enthesis (ENT) to the myotendinous junction (MTJ) at 42 days post-injection. E–G) SMA9+ and SMA9/ScxGFP+ cells are found at the muscle-tendon interface and in the perimysium (PM) of the extensor digiti minimi (EDM) tendon at 21 days. Dotted lines in E–G refer to orthogonal slices in the x–y, x–z, and y–z planes. SMA9/ScxGFP+ tendon fibroblasts and SMA9+ paratenon cells and muscle fibers are found at the MTJ of the Achilles tendon (H–J) 42 days. H&I are optical slices at the tendon surface and 30 µm deep in the AT, respectively (J is 3D reconstruction of H and I). All images were taken using two photon microscopy. Blue – second harmonic generation (SHG) for collagen. Scale bars = 100 µm. Anatomical locations of images can be found in figure S1.

**Figure 3:**
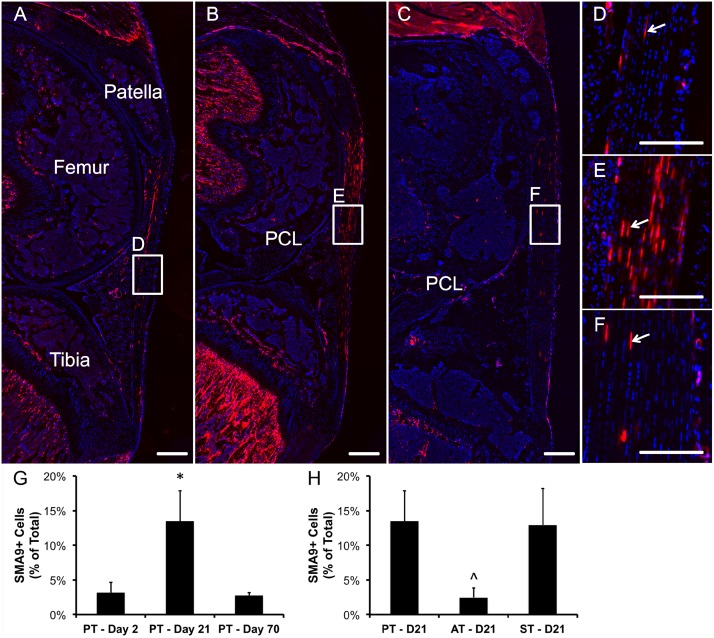
SMA9 model transiently labels an amplifying resident progenitor population in the tendon midsubstance during growth. A pulse chase experiment where two tamoxifen injections were given on consecutive days to 3–4 week old mice revealed that the initial number (3.1±1.5% of total) of resident SMA9+ cells (red, arrows) in the tendon midsubstance at 2 days (A,D,G) following injection expanded over 4-fold to 13.5±4.4% at 21 days (B,E,G) and then reduced to day 2 levels by 70 days (C,F,G). Trabecular bone in the tibia shows a similar trend, while muscle fibers in the quadriceps were labeled at later time points (C). No SMA9+ cells were found in the fibrocartilage of the enthesis or ligaments in the knee at any time point. In addition, the number of expanded cells on day 21 was significantly higher in the patellar (PT) and supraspinatus (ST) tendons compared to the achilles (AT) tendon (H, p<0.05). Blue – dapi counterstained nuclei. PCL – posterior cruciate ligament. Scale bars = 250 µm. *significantly different than other time points, ∧significantly different than other tendons. Error bars denote ± SD.

The resident tendon progenitors that contribute to postnatal growth and normal cell turnover are not well characterized. Lineage tracing of SMACre+ cells in 3–4 week-old SMA9 and SMA9-ScxGFP mice was determined at 21 and 42 days post tamoxifen activation (Figs. 1–2; anatomical locations for figures 1 and 2 can be found in figure S1). The labeled populations were visualized in three dimensions using two photon microscopy and defined by anatomical location, cell morphology, and/or GFP reporter expression. Four distinct populations of cells in and around tendons were identified: 1) circumferentially oriented, ring-like smooth muscle cells surrounded by adventitial collagen (as seen by second harmonic generation signal in blue) in larger vessels outside the tendon (Fig. 1A), 2) perivascular cells on the surface of smaller vessels outside the tendon (Fig. 1A–B,G), 3) cells in the paratenon (Fig. 1B, 2H), tendon sheath (Fig. 1D), or retinaculum (Fig. 1G) surrounding the tendon body, and 4) elongated SMA9-ScxGFP+ cells oriented between collagen fibers within the tendon midsubstance (Fig. 1C,F,H). All four of these populations are found in various limb tendons investigated in this study including the patellar, Achilles, superficial digital flexor (SDF), supraspinatus, and flexor/extensor tendons in the wrist at 21 and 42 days post-injection (Figs. 1–2).

SMA9+ cells in population 3 are either 1) situated within circumferentially oriented collagen fibers in the paratenon (Fig. 2H, S2), tendon sheath (Fig. 1D), or retinaculum (Fig. 1G) or 2) residing on the outer surface of the tendon proper just beneath the collagen fibers of the paratenon (Fig. 1B,E). The cells on the tendon surface have a flattened morphology while cells within the peritendinous structures are more elongated and orientated along collagen fibers. Cells within population 3 are primarily ScxGFP negative unlike cells in population 4 within the tendon that are both SMA9+ and ScxGFP+ at all time points investigated in this study (Figs. 1F,H and 2C,I), albeit the intensity of the ScxGFP signal varies from cell to cell. These cells are highly aligned along the tendon axis and are situated between densely packed collagen fibers.

In addition to cells within and around the tendon midsubstance, SMA9+ cells were found at the myotendinous junction (MTJ) (Fig. 2). Elongated SMA9/ScxGFP+ cells exist between collagen fibers of the tendon body distal to the MTJ (Fig. 2D,I). A combination of SMA9/ScxGFP+ and SMA9+ cells reside on the tendon surface (Fig. 2H) near the MTJ and in the perimysium surrounding the muscle that runs continuously with the tendon sheath, as seen in the EDM tendon (Fig. 2F–G). SMA9+ muscle fibers are observed at later time points following tamoxifen injection (Fig. 2H–J).

### SMA9 Cells in the Tendon Midsubstance are an Amplifying Resident Progenitor Population

Following the establishment that SMA9+ cells are found in 4 distinct populations in and around the tendon, we next questioned whether the cells in the body of the tendon (population 4) are an amplifying resident progenitor population that expands during growth. Growing mice (3–4 week-old) were injected on consecutive days with tamoxifen and the patellar tendons were assessed at 2, 21, and 70 days post-injection. On day 2, SMA9+ cells are found in all four populations described in the previous section (Fig. 3A,D). The cells within the tendon midsubstance are dispersed throughout the volume of the patellar tendon, constituting 3.1±1.5% of total cells (Fig. 3G), and they are all Scx+ (Fig. S3). By day 21, the population at 2 days amplifies by over 4-fold to 13.5±4.4% of cells in the tendon midsubstance (p<0.05; Fig. 3B,E,G). These cells appear to be dispersed throughout the tendon volume and there is little evidence of clonal expansion except within linear cell arrays, which show multiple labeled cells in succession. This pattern is not consistent however as seen in figure 1F where the SMA9+ cells alternate within the linear array. It also appears that this expanded population came from the cells labeled within the tendon body on day 2 and not cells within the paratenon on day 2, as there is often space between the internal population and tendon surface that did not contain SMA9+ cells (Fig. S4). When the chase is assessed on 70 days following injection (Fig. 3C,F), the number of cells in the tendon midsubstance diminishes to day two levels (2.8±0.4%, Fig. 3G), suggesting that the SMA9 model transiently labels an amplifying progenitor source within the body of the tendon during growth.

While these cells have the ability to expand during growth within the patellar tendon, other tendons show varying levels of expansion. When comparing the number of SMA9+ cells at day 21 within the patellar, Achilles, and supraspinatus tendon, we find that the expansion of SMA9 cells in the Achilles (2.5±1.3% of total) is significantly reduced compared to the other tendons (p<0.05; Fig. 3H). However, the number of SMA9 cells within the patellar and supraspinatus tendons was comparable (13.5±4.4% and 12.9±5.3, respectively).

### SMA9 Cells do not Contribute to Fibrocartilage in the Tendon-to-Bone Insertion Site or Ligaments

While SMA9+ cells are found throughout the tendon midsubstance and myotendinous junction at all time points, there are no SMA9+ cells in the fibrocartilage of the tendon-to-bone insertion (Fig. 3A–C). This is true in every tendon evaluated in this study and at every time point. There are also no SMA9+ cells in the cruciate and collateral ligaments of the knee (see PCL in Fig. 3B–C).

### GDF5-9 Model Labels all Cells in Ligaments and Regions of Tendon near Bone

Because SMA9+ cells are not found within the fibrocartilage of the tendon enthesis or within the ligaments of the knee and developmental studies show that distinct sets of progenitors form the enthesis vs the midsubstance [2], we investigated whether GDF5+ cells originating from the joint interzone would contribute to these regions. We crossed GDF5Cre mice with Ai9 reporter mice to demonstrate whether these regions came from Gdf5-expressing cells. These mice were analyzed at P0 (during tendon maturation) and P56 (during later stages of tendon growth). We found that GDF5 labels a population of cells (GDF5-9+) that extends from the tendon midsubstance, through the enthesis, and into the underlying bone.

**Figure 4:**
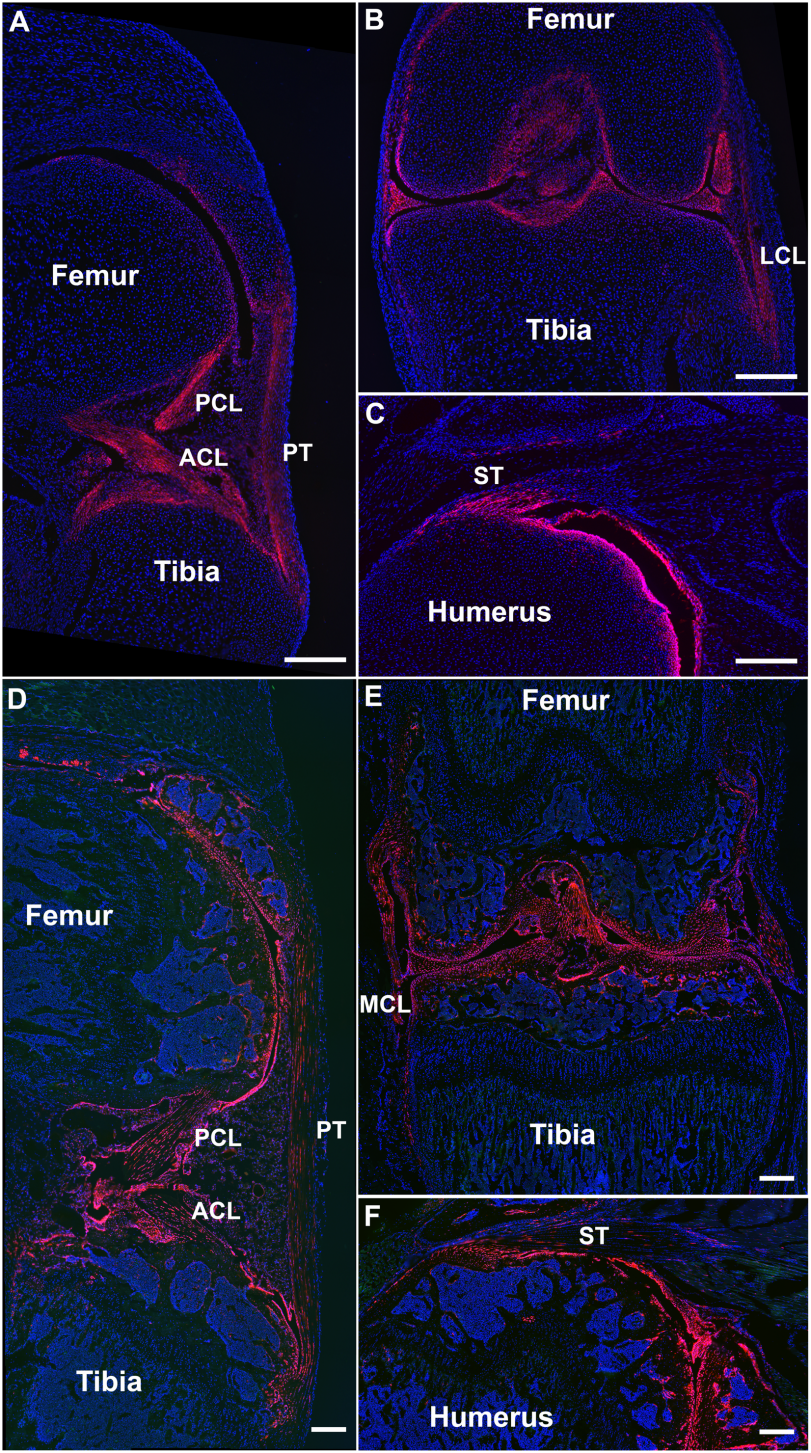
Unlike the SMA9 model, GDF5-9 cells are found within the fibrocartilage of the tendon enthesis and throughout the length of ligaments. GDF5-9+ cells are expressed in cells within the cruciate ligaments (ACL, PCL) from bone to bone (A,B,D,E). GDF5-9+ cells are concentrated near the enthesis of the supraspinatus tendon (ST) and do not extend along the length of the midsubstance (C,F). However, GDF5-9+ cells are found along the posterior half of the PT (A,D). GDF5-9 cells are also found within articular cartilage, menisci, collateral ligaments, synovium, and epiphyseal bone in the knee. These expression patterns are similar between the ages of P0 (A–C) and P56 (D–F) except the epiphyseal bone is not labeled at P0, which is before secondary ossification. Blue – dapi counterstained nuclei. LCL – lateral collateral ligament, MCL – medial collateral ligament. Scale bars = 250 µm.

During these different stages of tendon and joint development, GDF5-9+ cells are found within the fibrocartilage of the tendon enthesis of the patellar and supraspinatus tendons (Fig. 4A,C,D,F). A portion of cells isolated to the posterior half of the patellar tendon midsubstance is also GDF5-9+ (Fig. 4A,D). In addition, all ligamentous cells spanning from bone to bone are GDF5-9+ in the cruciate and collateral ligaments of the knee (Fig. 4A,B,D,E). There are also GDF5-9+ cells within the articular cartilage and within the epiphyseal bone but they were not found within the residual growth plate or the bone beneath the growth plate.

### SMA9 Resident Progenitors are the Main Contributors to Patellar Tendon Healing

**Figure 5:**
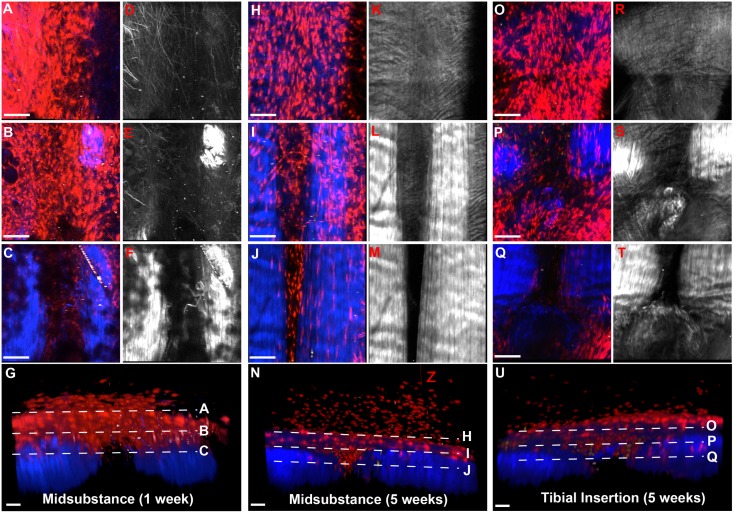
SMA9+ progenitors form a collagenous bridge over the anterior surface of the PT defect. Two photon imaging shows that SMA9+ cells within the thickened paratenon synthesize collagen (SHG signal in blue and grey) as they form the paratenon bridge. These cells originate from the medial and lateral tendon struts (G,N) and also from the anterior aspect of the tibia (O,P) and patella (data not shown). While these cells originate from the paratenon and vasculature, they infiltrate the adjacent struts over time (J). The collagen in the bridge matures from 1 week (A–G) to 5 weeks (H–U). G,N,U) 3D reconstructions in the axial view depicting the 3 levels in the anterior view (A–F, H–M, O–T). Scale bars = 100 µm.

**Figure 6:**
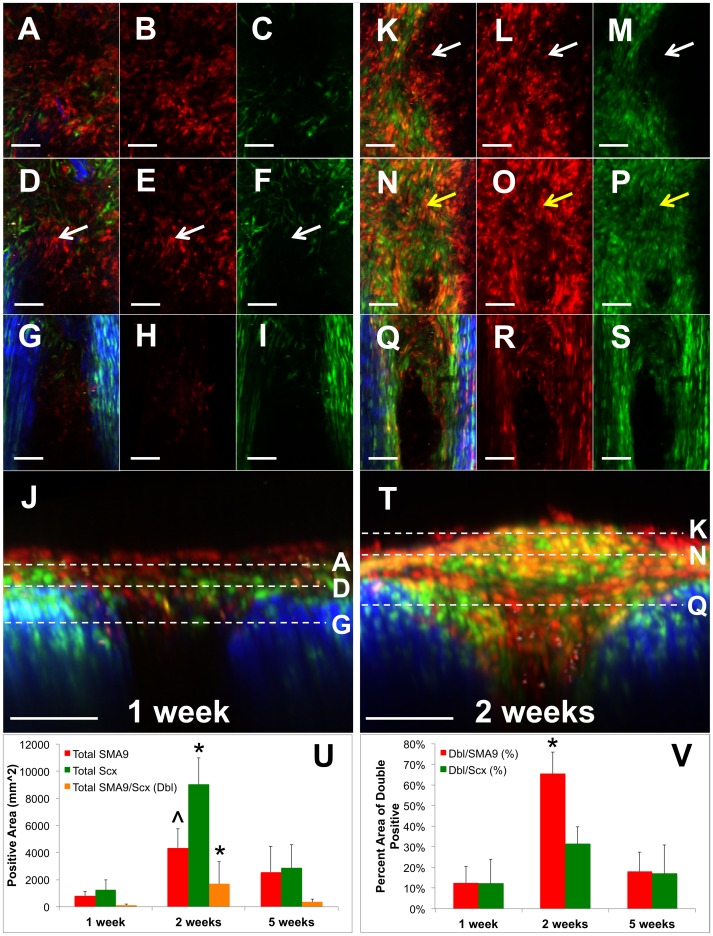
SMA9+ progenitors in the expanded paratenon differentiate into ScxGFP+ cells at 2 weeks post-injury. SMA9+ cells (white arrows, D–F) at one week post-injury (A–J) are predominately negative for ScxGFP as seen in two photon image stacks in SMA9-ScxGFP mice. However, these cells differentiate into ScxGFP+ cells (yellow arrows, N–P,V) at 2 weeks (K–T) within the collagenous regions of the paratenon bridge while SMA9+ only cells still remain on the anterior surface (white arrows, K–M). By 5 weeks, there is reduced overlap in SMA9 and ScxGFP expression (V). J,T) Axial views depicting the 3 levels in the anterior views (A–J, K–T) of the defects at 1 and 2 weeks, respectively. U) Total area of SMA9 and ScxGFP positive signal at 1, 2, and 5 weeks. V) SMA9-ScxGFP double positive area normalized to either total SMA9 or ScxGFP area, respectively. Scale bars = 100 µm. *significantly different than 1 and 5 weeks, ∧significantly different than 1 week (p<0.05). Error bars denote ± SD. Full z-stacks can be found in Video S1.

Since SMA9 progenitor cells were found to contribute to cell turnover during tendon growth, we next determined whether they contribute to tendon healing in the adult. We utilized a full-length, central-third patellar tendon defect injury used previously [20], [25] to examine the reparative potential of SMA9+ cells in the paratenon and tendon midsubstance. One week following injury, there is notable expansion of SMA9+ cells in the thickened paratenon, which forms in response to injury (Fig. 5A–G and S5A,E,I). The SMA9+ paratenon cells are initially found in the medial and lateral surfaces of the retinaculum (S5A,E,I) as well as on the surfaces of the tibia and patella (Fig. 5O,P). These SMA9+ cells expand to form an anterior bridge over the defect space by 2 weeks following injury (Fig. S5B,F,J and 6K–T). SMA9+ cells originating in the paratenon also infiltrate regions of the adjacent tendon struts as they remodel following injury (Fig. 5J, 6Q, and S5J,K). The thickened paratenon and remodeling regions of the struts are rich in tenascin-C (Fig. S5E–G in green), which forms a provisional matrix during healing.

The paratenon cells in the anterior bridge synthesize collagen fibers that are aligned perpendicular to the tendon axis over the adjacent struts as the cells expand from the anterior surfaces of the medial and lateral retinaculum (Fig. 5K,L). The collagen within the bridge consists of loose, small-diameter collagen fibers with poor organization at 1 week (Fig. 5D,E). By 5 weeks, the collagen matures in the bridge with thicker, densely packed fibers (Fig. 5K). However, the packing density is reduced compared to normal tendon or the adjacent struts (Fig. 5M).

A minimal number of SMA9+ cells are found in the contralateral, uninjured tendons (Fig. S5D,H,L). There are SMA9+ cells in the paratenon and tendon midsubstance, along with perivascular cells in small vessels outside of the tendon (Fig. S5L). However, the cells in the paratenon and tendon midsubstance did not expand over the 5 week time frame in these adult animals (Fig. S5H).

### SMA9+ Progenitors at 1 Week Post-injury Differentiate into ScxGFP+ Cells at 2 Weeks Post-injury

While SMA9+ cells within the paratenon expand to form a collagenous bridge over the defect space, we do not know if these cells differentiate into ScxGFP+ tenogenic fibroblasts or are non-specific scar fibroblasts. Therefore, we injured SMA9-ScxGFP triple transgenic mice. At 1 week following injury, there is a mixture of SMA9+ (white arrows, Fig. 6D–F, Video S1) and ScxGFP+ cells within the paratenon bridge; however, only 12.5±8.0% of these cells are double positive (Fig. 6A–J,V). At 2 weeks when the collagen within the bridge matures, the SMA9+ cells within the collagen are also ScxGFP+ (yellow arrows, Fig. 6N–P) while SMA9+ cells on the anterior surface above the mature collagen are still ScxGFP negative (white arrows, Fig. 6K–M). Taking these two populations into account, 65.5±10.6% of the total SMA9+ cells are SMA9-ScxGFP double positive at 2 weeks (Fig. 6V), which is significantly greater than at one week (p<0.05). As the paratenon bridge continues to mature at 5 weeks, there is reduced overlap in SMA9 and ScxGFP expression (18.1±9.3%).

## Discussion

Tendon is a composite structure with unique developmental and mechanical characteristics. It connects two tissues with distinct embryological origins: 1) bone from the sclerotome and 2) muscle from the myotome. The coordinated patterning during embryonic development involves separate progenitor pools to create the enthesis, tendon midsubstance and myotendinous junction [2], [3]. Once developed, tendons need to transmit tremendous contraction forces generated in the muscle down to the bone to enable skeletal movement. This transition is mechanically demanding as muscle and bone have orders of magnitude differences in mechanical properties, thus the tendon must be stiff enough to efficiently transfer load with minimal strain but also must integrate with these mechanically distinct structures in a fashion that reduces the stress concentrations at the interfaces. In addition, the tendon needs to adapt to the somatic growth of the individual both in terms of length and the increasing load it has to transmit secondary to a larger body size and increased utilization. Defining the cellular contributions to the tendon and its interaction with bone and muscle will be fundamental to understanding the molecular basis of this complex process.

Tendon has embryonic origins within Scx+ progenitors that reside in the region of the sclerotome adjacent to the myotome, known as the syndetome [1]. As the tendon condenses, Scx+/Sox9+ cells form a protrusion of fibrocartilage that anchors the tendon to bone (enthesis) [2], [3]. Concurrently, Scx+ progenitors at the MTJ synthesize collagen fibrils that attach to actin filaments within the sarcolemma to form the muscle attachment [26]. Once the enthesis-MTJ unit is formed, the tendon begins to lengthen to accommodate the somatic growth and to increase tendon fibril diameter to transmit greater mechanical loads. These morphologic changes suggest that both linear and appositional positioning of the progenitor cells is a requirement for normal somatic growth. Scx is required for proper tendon differentiation as it is initially expressed in an early progenitor and remains active throughout differentiation. Unfortunately, this means that Scx becomes less useful to label a progenitor population in a growing tendon as it is expressed in nearly all cells within the tendon body. Therefore, new markers are needed to characterize subtleties within the tendon cell population that contribute to tendon growth and maturation.

Our study has demonstrated that the SMA9 model marks an amplifying progenitor population capable of generating tendon cells, just as it does other mesenchymal tissues [9]–[12]. In the weanling mice that we studied, these cells arise within the body of the tendon and do not appear to migrate into the tendon from a peritendinous site (Fig. S4). The amplifications of these cells, which has not been seen previously using proliferation markers such as BrdU, is transient as the number of SMA9+ cells reduces to baseline levels after 10 weeks of expansion (Fig. 3G). Thus the population of SMA9+ cells is gradually replaced by unmarked cells as the tissue continues to grow in length and maturity. However, further characterization of these cells is needed to understand their phenotype and how to regulate their differentiation, especially since immunostaining for αSMA shows minimal expression in these cells (data not shown). Therefore, our future studies will isolate the SMA9+ cells to develop an expression profile for these early progenitors to characterize their phenotype and identify new and possibly improved markers.

Interestingly, the expansion of SMA9 resident progenitors differs from tendon to tendon. In fact, the number of SMA9+ cells at 21 days following injection is significantly reduced in the Achilles tendon compared to the patellar and supraspinatus tendons (Fig. 3H), while numbers at 2 days were comparable amongst these tendons (data not shown). The reduced expansion seen in the Achilles midsubstance also correlates with reduced mineral apposition rate of the mineralized fibrocartilage of the enthesis (unpublished data) during this time period. The cause of these differences is unknown and suggests that the cell and matrix turnover of the Achilles is reduced at this age of growth, which is puzzling as the Achilles continues to grow linearly with the tibia during this period. It is possible that the mechanism of growth in the Achilles may be different from other tendons.

While SMA9+ progenitors contribute to the tendon midsubstance, they do not contribute to cells within the fibrocartilage of the tendon insertion, which are derived from GDF5-9+ cells. GDF5-9+ cells contribute to the enthesis along with a segment of the tendon midsubstance (Fig. 4). This lineage driver indicates that the enthesis may develop from the interzone cells that generate the structures within the forming joint space [15]. This observation is consistent with previous work demonstrating that Scx+/Sox9+ progenitors, distinct from the primary cartilage, form the enthesis during embryogenesis, while Scx+ only progenitors form the midsubstance [2]. These findings lend credence to the composite nature of tendon and the heterogeneous origin of cells within these regions.

One of the interesting outcomes of this study was that the patellar tendon was the only tendon analyzed that had GDF5-9+ cells along the whole tendon length, spanning from patella to tibia on the posterior side of the tendon. This contributes to the debate on whether the patellar tendon is indeed a tendon or a ligament. Anatomical, architectural, and cell lineage analysis suggests that it may be a mixture of both. By the purest definition, the patellar tendon is a ligament as it attaches bone (patella) to bone (tibia). However, the patella is a specialized sesamoid bone that forms from a pool of Scx+ progenitor cells during development, as knocking out Sox9 in Scx-expressing cells will prevent the patella from forming at all [2]. If the SMA9 model marks a consistent progenitor pool from tendon to tendon but doesn’t label a pool within ligaments, this suggests that the patellar tendon is more tendon-like as well. However, GDF5, which is highly expressed in ligaments, is also expressed on the posterior half of the patellar tendon. Therefore, the patellar tendon has unique tendinous and ligamentous properties.

**Figure 7:**
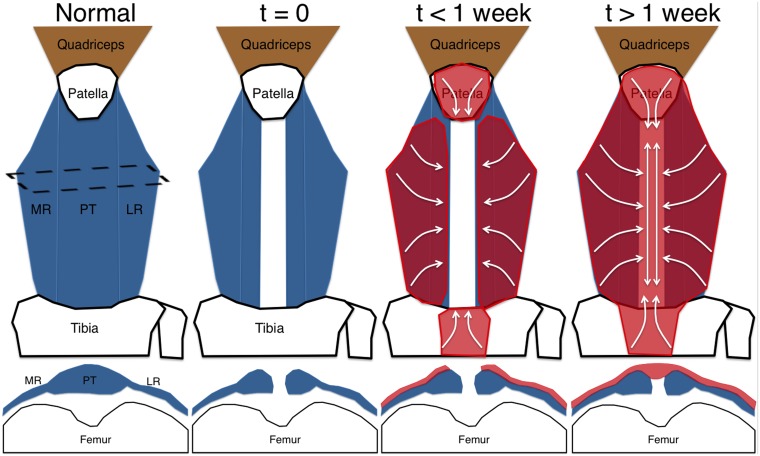
SMA9+ cells from the paratenon/periosteum of the adjacent tendon and bone are the main contributors to PT healing. Schematic depicts the origin and directional expansion of cells that contribute to PT defect healing. These cells originate within the paratenon in the tendon and retinaculum of the medial/lateral aspects as well as paratenon/periosteum over the proximal and distal bones. As the cells form the anterior bridge over the defect space, they begin to align along the tendon axis. MR – medial retinaculum, LR – lateral retinaculum.

In addition to SMA9+ progenitors cells’ contribution to tendon growth, SMA9+ progenitors amplify following injury and are the main contributors to healing of the central PT defect (Fig. 5–7). These cells are found within an expanded paratenon that extends from the adjacent retinaculum to span the defect space (Fig. 7). In addition, cells from the periosteum on the anterior surface of the tibia also expand in response to the tendon injury and work to fill the space near the tibial insertion (Fig. 5O–U). Perivascular cells within the expanded paratenon are also SMA9+ (Fig. 5I). Unfortunately, we cannot discriminate between the relative contribution of the paratenon and perivascular progenitors in this SMA9 model, which is a limitation that requires additional markers to isolate the perivascular from the paratenon populations.

As the repair process matures, the cells over the defect space acquire an elongated morphology in parallel with the native tendon and express the ScxGFP reporter (Fig. 6N). However few of these cells become incorporated into the native tendon or fuse with the ligated ends of the central tendon defect (Fig. 5Q,T). Instead this new peritendinous tissue is distinctly separate from the adjacent tendon and matures with time, leading to improved mechanical properties [25]. Interestingly, the number of SMA9-ScxGFP double-positive cells in the paratenon reduces from 2 to 5 weeks post-injury (Fig. 6V). This reduction could be caused by cell turnover as there are fewer SMA9 cells at 5 weeks (Fig. 6U) or these SMA9+ cells that were ScxGFP+ at 2 weeks are ScxGFP negative at 5 weeks. We have previously shown that the mechanical improvement of the healing tissue plateaus at 5 weeks [25], which may suggest that maintaining Scx expression in these cells for longer periods may lead to continued improvement and is a focus of our future strategies.

We also see a similar mechanism of healing in response to long bone fracture where SMA9+ progenitors within the periosteum are the main contributors to the callus formation [9], [27]. These data combined suggest that adventitial structures surrounding both tendon and bone, which are also highly vascularized, may hold a reserve of SMA9+ progenitors that activate and amplify in response to injury. A better understanding of the phenotype of these progenitors and how to promote their differentiation during healing will be critical for improving repair outcomes of these injuries. Future studies will investigate these mechanisms using the SMA9 model.

This study underlines the complexity and heterogeneity of cells that initially form the midsubstance of tendons and subsequently participate in a reparative response to injury. Although both map to the similar SMA9+ progenitor sources, they appear to have different locations and capabilities. Models of regeneration in the intestine, hair follicle and hematopoietic system suggest that a tissue resident stem cell undergoes asymmetrical division in which the daughter cells have three potential fates: 1) return to a quiescent and mitotically inactive state, 2) enter a differentiated inactive state capable of rapid reactivation for formation of multiple cell types, or 3) enter a pool of transient amplifying cells with a constrained differentiation fate as seen with the SMA9 model during growth [28]. Stochastic factors that are inherent to the tissue niche probably control which option the daughter stem/progenitor cells choose. Genetic strategies to distinguish these three states have been developed for epithelial tissues and this will be the challenge for the skeletal biologist. Identifying cells within the tendon and adjacent tissues and learning how to manipulate their expansion and differentiation will be a requirement for developing reparative strategies that might lead to a fully functional and long-lived outcome.

## Supporting Information

Figure S1 **Anatomical locations of images in** **figures 1** **and** **2** **.** A) Posterior view of ankle depicting Achilles tendon (AT) with overlapping superficial digital flexor (SDF) tendon. B) Anterior view of patellar tendon (PT). C) Anterolateral view of flexor carpi ulnaris (FCU) tendon in the wrist. D) Superior view of supraspinatus tendon (ST) in shoulder following reflection of deltoid muscle and clavicle/acromion. E) Posterolateral view of extensor digiti minimi (EDM) tendon with adjacent extensor carpi ulnaris (ECU) tendon in the wrist. The area for each panel in figures 1 and 2 are denoted by black boxes. Panel E’ is high magnification view of the red box in panel E.
(TIFF)

Figure S2 **Paratenon cells within circumferentially oriented collagen fibers are SMA9+.** Composite two photon image of SMA9/ScxGFP Achilles tendon at 42 days post-injection. Arrows point to SMA9+ cell situated on collagen within the paratenon, which wraps around the tendon surface. Red – SMA9+ cells, Green – ScxGFP+ cells, Yellow/Orange – SMA9+/ScxGFP+ cells, Blue – SHG for collagen.
(TIFF)

Figure S3 **Two days following tamoxifen injection, SMA9+ only cells are found within the paratenon (arrow heads) on the tendon surface while SMA9+/ScxGFP+ cells are found within the tendon body (arrows).** A–B) 3D reconstruction in the anterior view of the patellar tendon where the tendon axis runs in the y-direction. C–D) The 3D reconstruction from A & B was rotated to an anterior-lateral view. Red – SMA9+ cells, Green – ScxGFP+ cells, Yellow/Orange – SMA9+/ScxGFP+ cells, Blue – SHG for collagen.
(TIFF)

Figure S4 **There is often a gap between population 3 on the tendon surface and population 4 within the tendon body.** A–B) Thin section in the transverse orientation of the PT of a SMA9/ScxGFP mouse. C–D) 3D reconstruction in the anterior view of the patellar tendon where the tendon axis runs in the y-direction. E–F) 3D reconstruction in the lateral view. G–H) 3D reconstruction in the axial view. Red – SMA9+ cells, Green – ScxGFP+ cells, Yellow/Orange – SMA9+/ScxGFP+ cells, Blue – cell nuclei (A–B) and SHG for collagen (C,E,G).
(TIFF)

Figure S5 **SMA9+ paratenon cells contribute to patellar tendon defect healing.** Full-length, central PT defects were created in adult mice. Tamoxifen injections were delivered on the day of surgery and the day following. SMA9+ progenitors within the paratenon expand in response to injury, leading to a thickened paratenon compared to normal tendon (D,H,L). By one week (A,E,I), SMA9+ cells from the paratenon and perivasculature have reached the defect space. A bridge over the anterior surface forms by 2 weeks (B,F,J) and matures at 5 weeks (C,G,K). Tenascin-C (green) is a major ECM component in this healing matrix where the SMA9+ cells are located. A–D are toluidine stained sections. Blue – dapi counterstained nuclei. Scale bars = 100 µm.
(TIFF)

Video S1 **Multiphoton z-stacks of PT defects at 1, 2, and 5 weeks in SMA9-ScxGFP mice.** 1st row – Red: SMA9, Green: ScxGFP, Blue: SHG for collagen. 2nd row – Red: SMA9, Green: ScxGFP. 3rd row – Gray: SHG for collagen. 4th row – Transverse view of rows 1–3. Dotted line refers to depth within tendon starting at anterior surface and moving deeper into tendon.
(AVI)
