# Supplementary figures and images for: Lineage Tracing of Resident Tendon Progenitor Cells during Growth and Natural Healing
Source: PLoS One. 2014 Apr 23;9(4):e96113. doi: 10.1371/journal.pone.0096113 (PMC3997569; doi:10.1371/journal.pone.0096113)

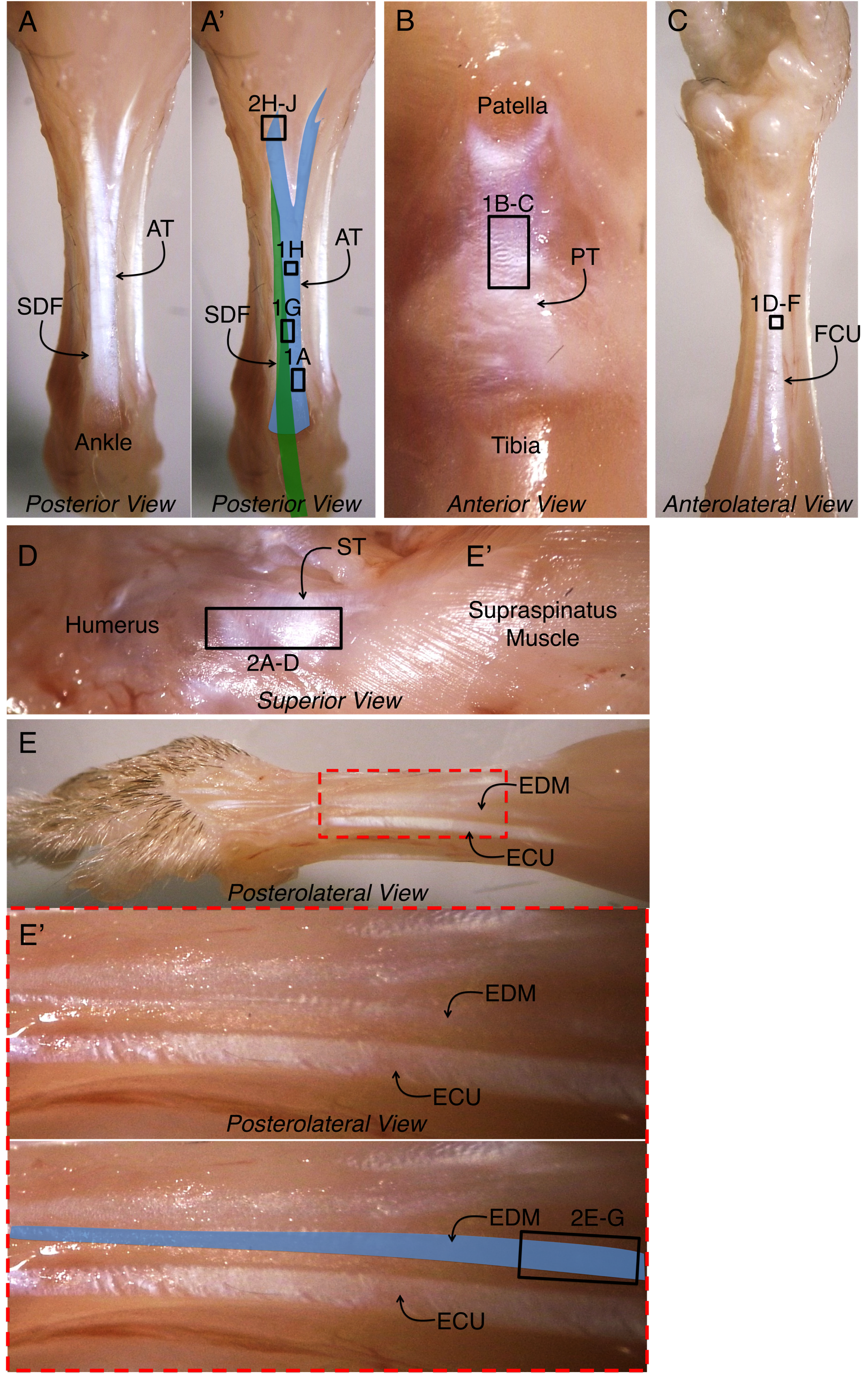

Supplement: Figure S1 — Anatomical locations of images in figures 1 and 2 . A) Posterior view of ankle depicting Achilles tendon (AT) with overlapping superficial digital flexor (SDF) tendon. B) Anterior view of patellar tendon (PT). C) Anterolateral view of flexor carpi ulnaris (FCU) tendon in the wrist. D) Superior view of supraspinatus tendon (ST) in shoulder following reflection of deltoid muscle and clavicle/acromion. E) Posterolateral view of extensor digiti minimi (EDM) tendon with adjacent extensor carpi ulnaris (ECU) tendon in the wrist. The area for each panel in figures 1 and 2 are denoted by black boxes. Panel E’ is high magnification view of the red box in panel E. (TIFF) [file pone.0096113.s001.tif]

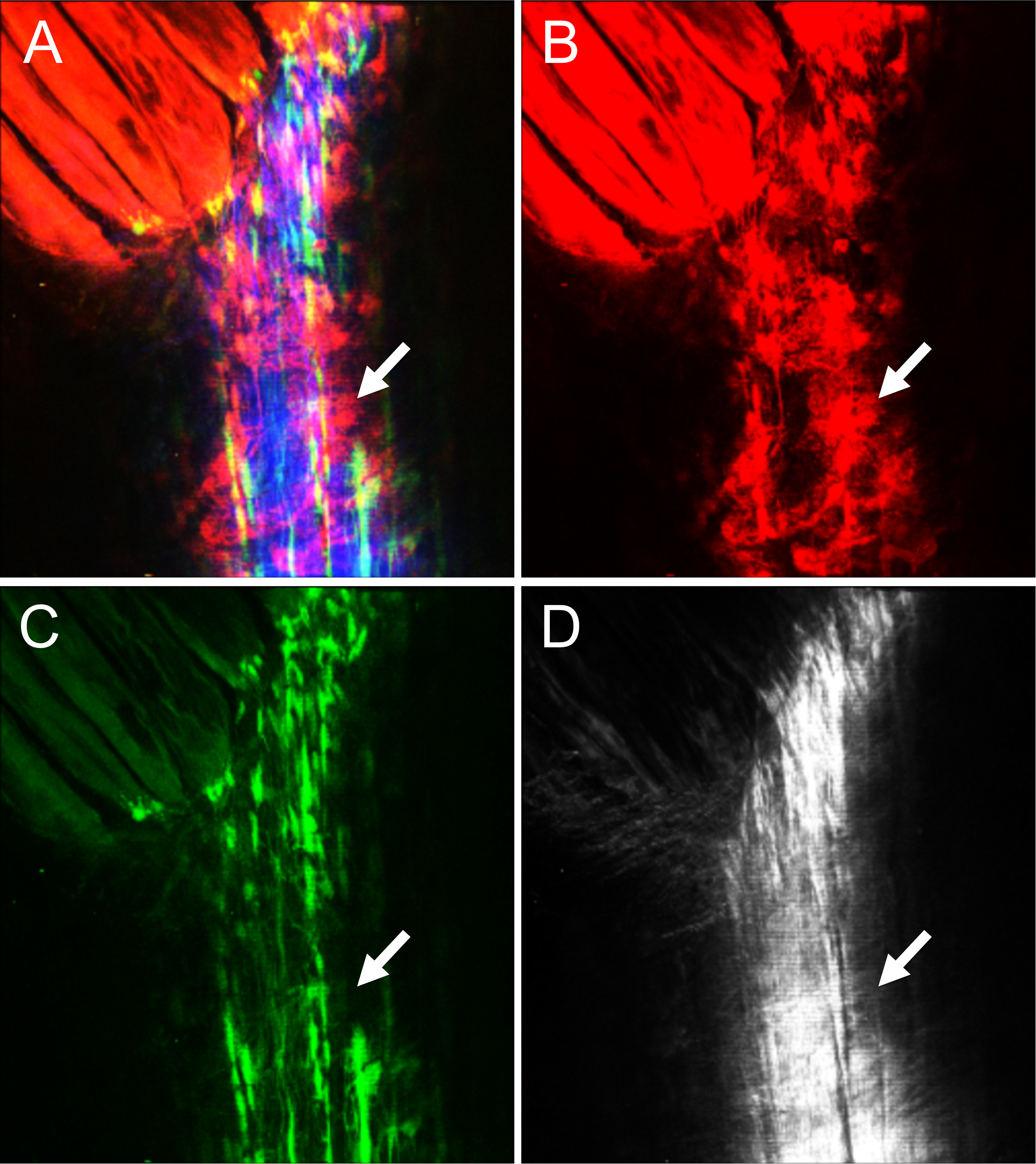

Supplement: Figure S2 — Paratenon cells within circumferentially oriented collagen fibers are SMA9+. Composite two photon image of SMA9/ScxGFP Achilles tendon at 42 days post-injection. Arrows point to SMA9+ cell situated on collagen within the paratenon, which wraps around the tendon surface. Red – SMA9+ cells, Green – ScxGFP+ cells, Yellow/Orange – SMA9+/ScxGFP+ cells, Blue – SHG for collagen. (TIFF) [file pone.0096113.s002.tif]

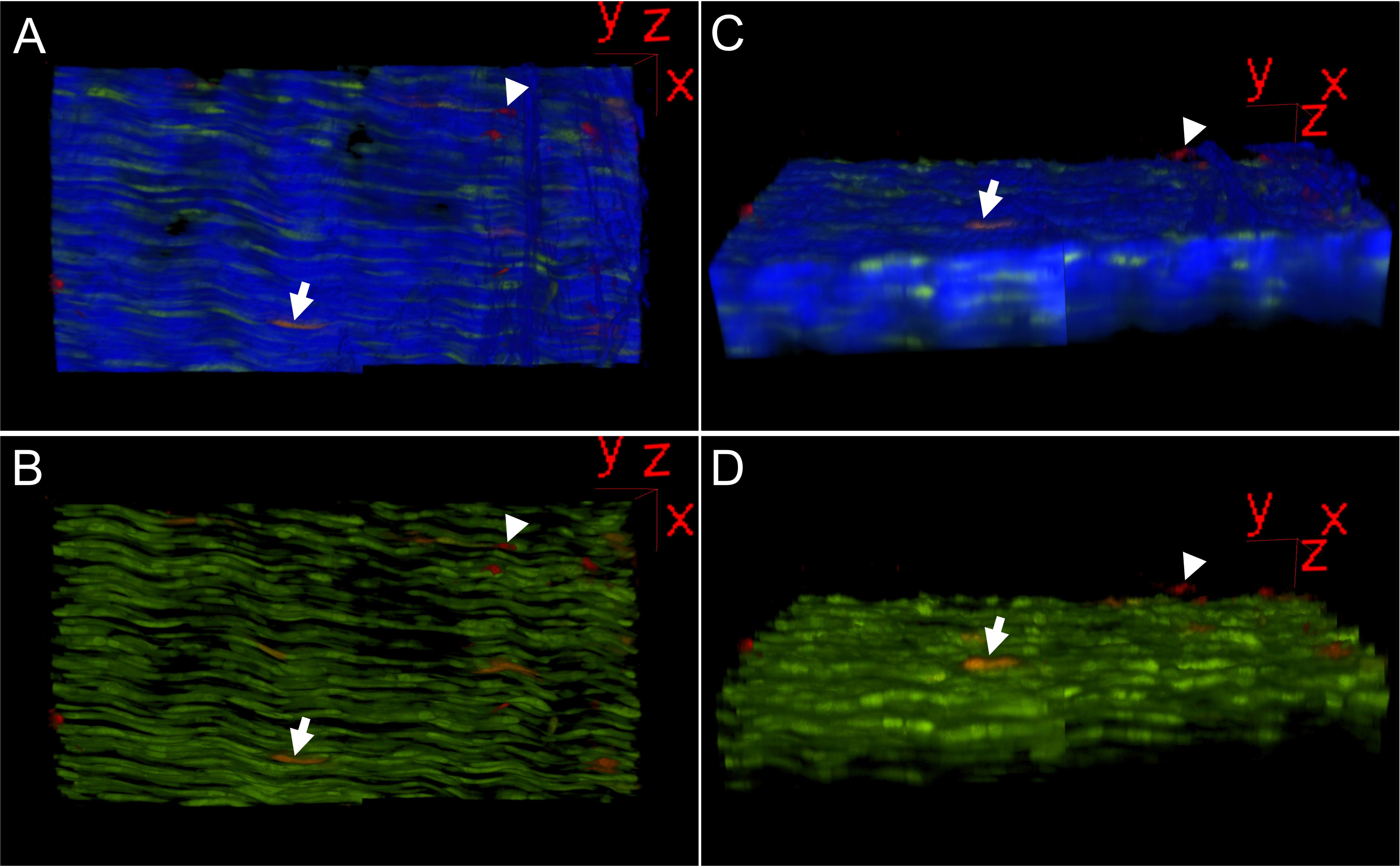

Supplement: Figure S3 — Two days following tamoxifen injection, SMA9+ only cells are found within the paratenon (arrow heads) on the tendon surface while SMA9+/ScxGFP+ cells are found within the tendon body (arrows). A–B) 3D reconstruction in the anterior view of the patellar tendon where the tendon axis runs in the y-direction. C–D) The 3D reconstruction from A & B was rotated to an anterior-lateral view. Red – SMA9+ cells, Green – ScxGFP+ cells, Yellow/Orange – SMA9+/ScxGFP+ cells, Blue – SHG for collagen. (TIFF) [file pone.0096113.s003.tif]

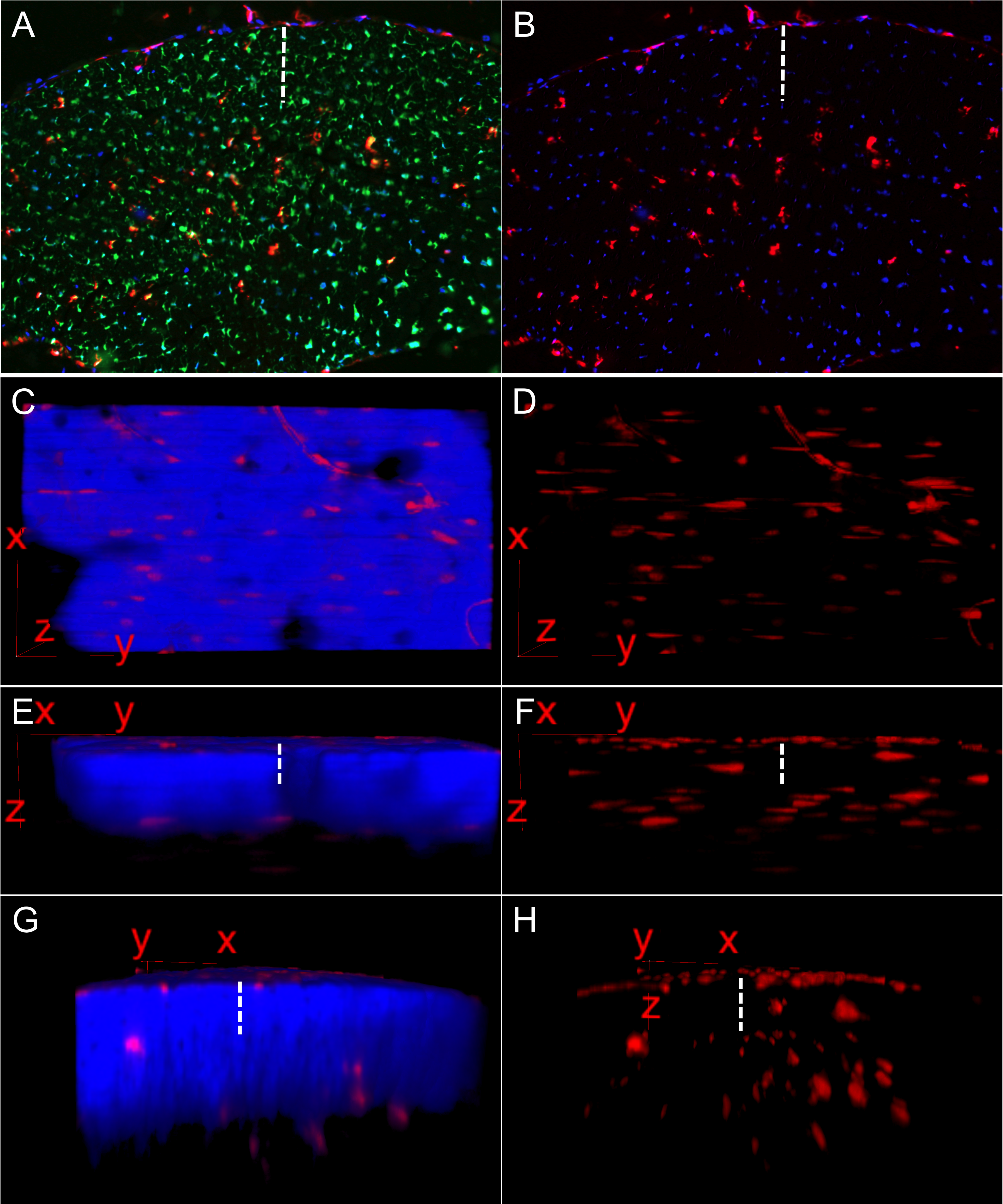

Supplement: Figure S4 — There is often a gap between population 3 on the tendon surface and population 4 within the tendon body. A–B) Thin section in the transverse orientation of the PT of a SMA9/ScxGFP mouse. C–D) 3D reconstruction in the anterior view of the patellar tendon where the tendon axis runs in the y-direction. E–F) 3D reconstruction in the lateral view. G–H) 3D reconstruction in the axial view. Red – SMA9+ cells, Green – ScxGFP+ cells, Yellow/Orange – SMA9+/ScxGFP+ cells, Blue – cell nuclei (A–B) and SHG for collagen (C,E,G). (TIFF) [file pone.0096113.s004.tif]

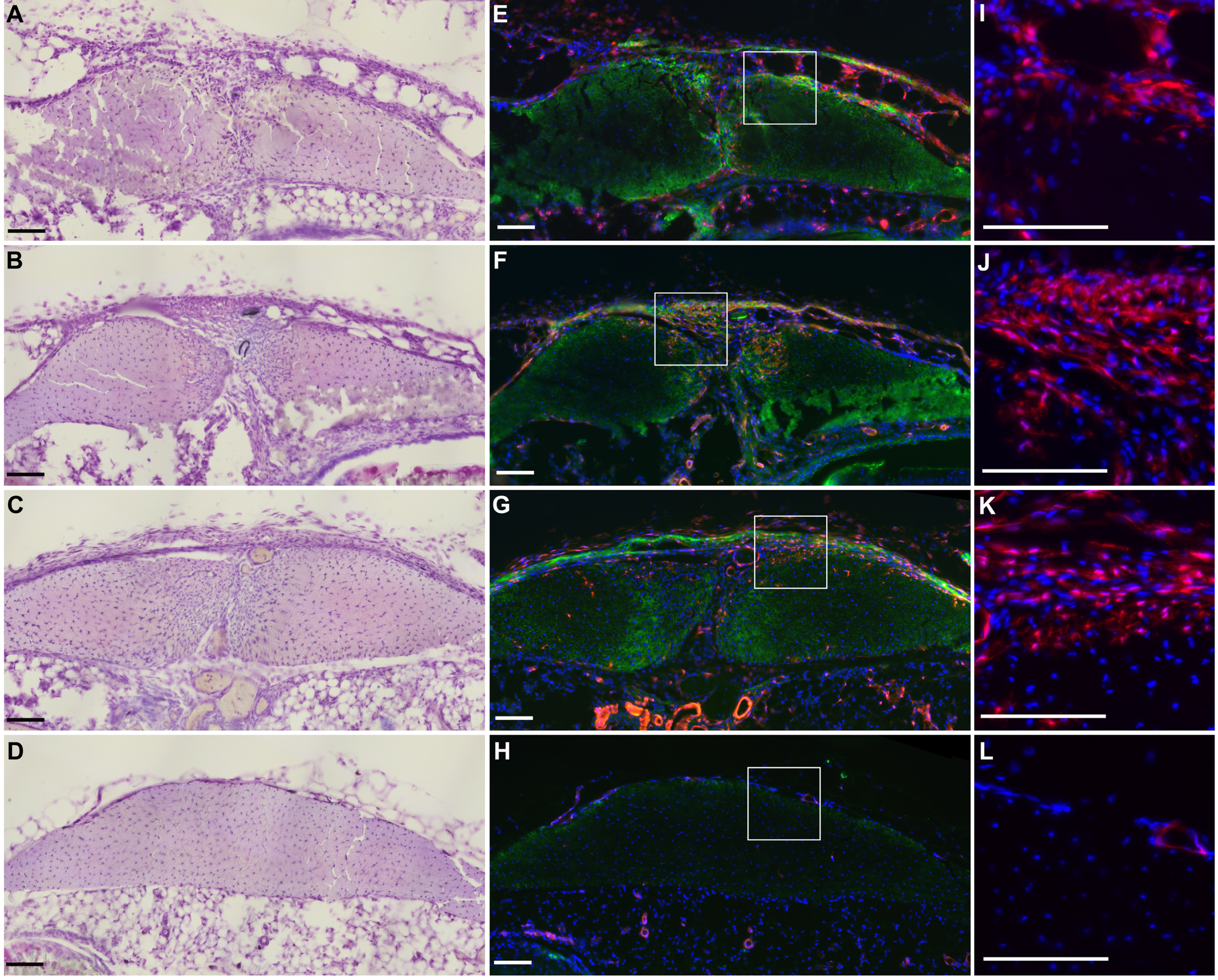

Supplement: Figure S5 — SMA9+ paratenon cells contribute to patellar tendon defect healing. Full-length, central PT defects were created in adult mice. Tamoxifen injections were delivered on the day of surgery and the day following. SMA9+ progenitors within the paratenon expand in response to injury, leading to a thickened paratenon compared to normal tendon (D,H,L). By one week (A,E,I), SMA9+ cells from the paratenon and perivasculature have reached the defect space. A bridge over the anterior surface forms by 2 weeks (B,F,J) and matures at 5 weeks (C,G,K). Tenascin-C (green) is a major ECM component in this healing matrix where the SMA9+ cells are located. A–D are toluidine stained sections. Blue – dapi counterstained nuclei. Scale bars = 100 µm. (TIFF) [file pone.0096113.s005.tif]
